# An Interesting Sequel of Pulmonary Tuberculosis

**DOI:** 10.7759/cureus.48928

**Published:** 2023-11-16

**Authors:** Bhumika Vaishnav, Ruchitha Pailla

**Affiliations:** 1 General Medicine, Dr. D. Y. Patil Medical College, Hospital & Research Centre, Pune, IND

**Keywords:** fibrosis, lung neoplasms, squamous cell, carcinoma, pulmonary, tuberculosis

## Abstract

Tuberculosis (TB), although a preventable and curable disease, accounts for a high burden of morbidity in developing countries like India. Lung scarring and damage is a common sequel of pulmonary tuberculosis (PTB). Here, we report an interesting case of a patient with a history of PTB diagnosed four years ago and on subsequent visits was diagnosed with squamous cell carcinoma of the lung at the site of the TB scar. The development of malignancy in the old PTB scar is a controversial yet often diagnosed sequel of PTB.

## Introduction

Tuberculosis (TB) is an age-old enemy of mankind. The estimated incidence of TB in India, for the year 2021, as reported by the World Health Organization (WHO), was 188/100,000 population [1]. It means that there is a sizeable number of people living in India, who were treated for pulmonary tuberculosis (PTB) in the past. Even after a successful treatment of PTB, the lung parenchyma may become scarred, leading to sequelae, such as bronchiectasis, cavitation, and chronic obstructive pulmonary disease [2]. Here, we present an interesting case of lung squamous cell carcinoma, which developed at the site of the scar of old PTB.

## Case presentation

A 56-year-old male, previously treated case of PTB, presented with complaints of breathlessness and right lower limb pain of one-day duration. He had no known addictions. He was diagnosed with right-lower-zone PTB four years ago, was sputum positive, and had completed six months of anti-tubercular treatment under the National TB Elimination Program (NTEP) and was declared cured after the treatment. He had complaints of acute onset dyspnea at rest for one day, not associated with chest pain, trauma, fever, foreign body inhalation, or palpitations. He also complained of right lower limb pain for one day before admission. He gave a history of weight loss of seven kilograms in the last two months. He had no history of smoking.

**Figure 1:**
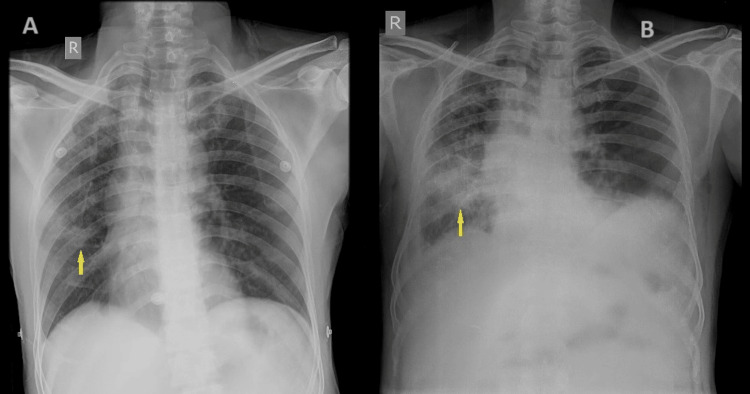
Chest X-ray A. Chest X-ray post-treatment for PTB done in 2019 shows right apical and mid-zone patchy changes of fibrosis. B. Large patch of consolidation in the right middle and lower lung zones showing spiculated margins and air bronchogram within and blunting of costophrenic angles with fissural extension – s/o bilateral pleural effusion.

On examination, the patient’s pulse rate was 124/minute, blood pressure was 140/100 mm of Hg, respiratory rate was 40/minute, and oxygen saturation on room air was 83%. He had right calf tenderness and pitting pedal edema up to the level of the right knee. The laboratory studies showed hemoglobin 11.5 g%, total leukocyte count 10.5 cells x 10^9 ^L/, platelet count 180x10^9^ /L, and D-dimer 8565 ng/ml, and renal and liver functions and serum electrolytes were normal. Figure 1 shows the chest X-rays of the patient. The first one done after the completion of the anti-tubercular treatment in 2019 was suggestive of right mid- and lower-zone fibrosis, and the second done during the current admission was suggestive of right mid-zone/lower-zone consolidation with bilateral pleural effusion. Sputum culture, Gram, and Ziehl-Neelsen stains were negative. Sputum cartridge-based nucleic acid amplification test (CB-NAAT) was negative for *Mycobacterium tuberculosis*. Ultrasound of the abdomen and pelvis showed mild ascites. Both lower limb venous Doppler showed partially compressible deep femoral vein with thrombus on the right side, suggestive of right lower limb deep vein thrombosis (DVT).

**Figure 2:**
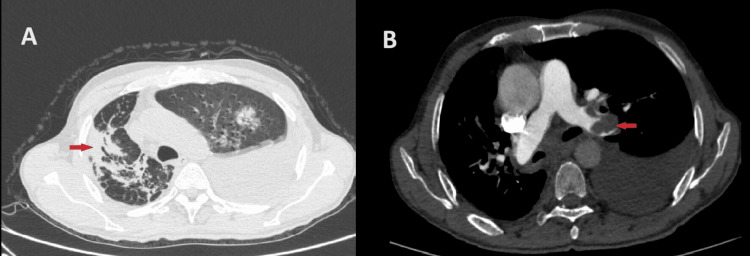
A. High-resolution computerized tomography (HRCT) showing a sub-pleural right-side pulmonary mass lesion of a likely neoplastic etiology with left-side pleural effusion. B. CT-pulmonary angiography (CTPA) showing thrombus in the left main pulmonary artery s/o PTE.

**Figure 3:**
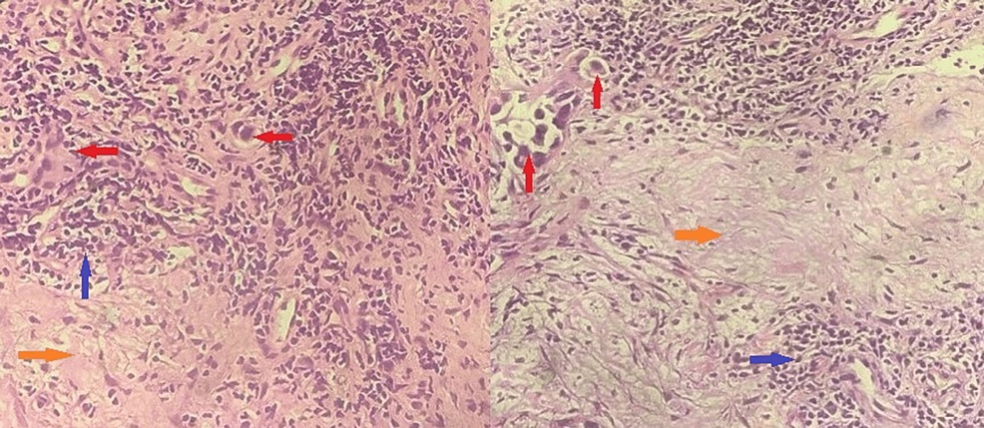
Lung biopsy histopathology (hematoxylin and eosin stain): pleomorphic cells and individual cell show hyperchromatic nuclei with prominent nucleoli and moderate eosinophilic cytoplasm (red arrow) and stromal reaction with areas of hyalinization (orange arrow) and inflammatory infiltrates (blue arrow) around the tumor.

Figure 2 shows the high-resolution computerized tomography (HRCT) and CT-pulmonary angiography (CTPA) images of a patient. The HRCT showed a sub-pleural right-side pulmonary mass lesion of a likely neoplastic etiology with left-side pleural effusion. CTPA showed bilateral pulmonary thromboembolism of the left main pulmonary and right middle lobe arteries. A CT-guided right lung biopsy was done. Histopathological examination of the lung tissue showed clumps of malignant squamous cells with stromal reaction, areas of hyalinized fibrosis, and anthracotic tissue suggestive of squamous cell carcinoma on a background of fibrous tissues (Figure 3). Thus, our final diagnosis was squamous cell lung carcinoma with bilateral pulmonary thromboembolism, right lower limb DVT in a known case of old PTB. The patient had DVT and PTE, which are known complications of lung carcinoma. Usually, smoking addiction is a risk factor for the development of both PTB and lung cancer. It is one of the most common positive confounding factors, which was not present in our patient. Thus, the patient had no other known risk factors for the development of lung cancer. As the lung mass was seen exactly at the same site as that of the old PTB, and it had a peripheral location with histopathological features suggestive of scarring within the malignant tissue, we diagnosed it as a scar carcinoma of the lung, post-PTB. Positron emission tomography (PET CT) was suggestive of a right-lower-zone fluorodeoxyglucose (FDG) avid lesion of size 28 x 14 mm with no metastatic lesions anywhere else in the body. The patient was started on palliative chemotherapy with carboplatin and paclitaxel planned for six cycles.

## Discussion

There are several reports of patients developing lung cancer at the site of old PTB scars [3-5]. The proposed theories for this association include the presence of chronic inflammation, scarring of lung parenchyma, and genetic damage caused by *Mycobacterium tuberculosis*, which is believed to initiate the process of carcinogenesis and eventually lead to lung cancer [3]. Adenocarcinoma is the most common histopathological type of lung cancer, followed by epidermoid carcinoma. Lung scar carcinoma was originally described by histopathologist Friederich in 1939 [6]. Although it is not included in the histological classification of lung cancer as per the International Association for the Study of Lung Cancer, many previous studies have suggested the presence of the entity of "scar carcinoma" [7,8]. The so-called scar carcinoma or “scarcinoma” is thought to arise from the scars of previous infections, inflammations, or parenchymal insults, such as fibrosis [9]. Lung fibrosis is a common sequel of PTB. Lung fibrosis is a response to a lung injury, where there is an accumulation of activated myofibroblasts and mesenchymal cells leading to the deposition of an extracellular matrix (ECM) [10]. A vicious cycle of autonomous worsening of this fibrosis process may occur due to an aberrant interaction between the epithelial and mesenchymal cells and the ECM. In lung neoplasms, the same process may continue with malignant transformation for the formation of stroma of the tumor, which is fundamental for cancer progression. Persistent lung inflammation and activation of mesenchymal cells due to PTB are believed to cause lung cancer by the same mechanism. It also leads to deoxyribonucleic acid (DNA) breakages, which is critical to carcinogenesis [10].

Moreover, the lateral genetic transfer of mycobacterial DNA into the bronchial epithelial cell DNA may cause neoplastic transformation [3]. Thus, malignant transformation in a previously healed TB scar of the lung is one of the rare sequels of PTB. 

India is a country with a high prevalence of PTB. Thus, there are many patients with lung scars due to previously treated or partially treated PTB who may have an ongoing inflammatory process in the lungs, which may predispose them to lung malignancy.

## Conclusions

As physicians, all must be aware of the possibility that old PTB may predispose patients to malignancy so that it can be diagnosed and treated in a timely manner. Unexplained weight loss after the treatment of TB with no detection of mycobacterium should raise the suspicion of scar carcinoma. A CT scan of the chest and lung tissue biopsy may be helpful investigations for the diagnosis.
